# Muscle morphology changes over 1-year in patients with rheumatoid arthritis: a prospective cohort study using muscle ultrasound

**DOI:** 10.1186/s42358-026-00529-z

**Published:** 2026-07-21

**Authors:** Leonardo Peterson dos Santos, Rafaela Cavalheiro do Esíprito Santo, Émerson Pena, Stephanie Pilotti, Daniel Nóbrega Moraes, André Luiz Silveira Mallmann, Lucas Denardi Dória, Gabriel Eduardo Grave, Vanessa Hax, Nicole Pamplona Bueno de Andrade, Claiton Viegas Brenol, Odirlei André Monticielo, Rafael Mendonça da Silva Chakr, Ricardo Machado Xavier

**Affiliations:** 1 Autoimmune Disease Laboratory, Federal University of Rio Grande do Sul, Hospital de Clínicas de Porto Alegre, R. Ramiro Barcelos, 2350, Porto Alegre, 90035-003 Brazil; 2 Rheumatology Service, Hospital de Clínicas de Porto Alegre, Porto Alegre, Brazil; 3 Health Research and Innovation Science Centre, Klaipėda University, Klaipėda, Lithuania; 4 Faculty of Medicine, Federal University of Rio Grande do Sul, Porto Alegre, Brazil

**Keywords:** Rheumatoid arthritis, Muscle mass, Ultrasound, Muscle strengtha, Functionality

## Abstract

**Background:**

Low muscle mass is associated with physical disability in rheumatoid arthritis (RA), highlighting the need for regular muscle assessment. Muscle ultrasound (MU) is a promising tool for evaluating muscle morphology, however, longitudinal studies in RA are lacking. This study aimed to evaluate changes in quadriceps muscle thickness and pennation angle using MU and identify predictors of these changes.

**Methods:**

This prospective observational cohort included women and men with RA. Muscle thickness and pennation angles of the rectus femoris (RF), vastus intermedius (VI), and vastus lateralis (VL) were assessed using MU. Disease activity was measured using the 28-joint Disease Activity Score based on C-reactive protein (DAS28-CRP), muscle strength by the handgrip test, physical performance by Short Physical Performance Battery (SPPB), physical disability by Health Assessment Questionnaire (HAQ), and physical activity by International Physical Activity Questionnaire (IPAQ). Paired t-tests, Pearson and Spearman correlations analyses, and multiple linear regression were conducted (p≤0.05).

**Result:**

Among 155 RA patients (88.4% women), 106 completed the 1-year follow-up. The baseline mean age was 58.7±9.5 years, median disease duration was 11.0 (6.0–20.0) years, and mean DAS28-CRP was 3.02±1.29. At follow-up, women exhibited a 7% reduction in VL, 6% in RF, and 4% in VI pennation angles (p≤0.05). Men showed a 39% increase in RF and a 29% in VI muscle thickness (p≤0.05). In women, lower baseline DAS28-CRP and higher muscle strength were correlated with increased muscle thickness (p≤0.05), with DAS28-CRP identified as a predictor of changes in muscle thickness (β=-0.058; CI95%,-0.114 to -0.003).

**Conclusion:**

RA women exhibited a decrease in pennation angle, while men had an increase in muscle thickness over time. Disease activity was a significant predictor of these changes in women, underscoring MU’s clinical utility.

**Supplementary Information:**

The online version contains supplementary material available at 10.1186/s42358-026-00529-z.

## Background

Patients with early and established rheumatoid arthritis (RA) demonstrate reduced muscle mass compared to age- and sex-matched healthy controls [1, 2]. Studies indicate that, even with adequate intensive treatment, RA patients have approximately 10% less appendicular lean mass than healthy controls [2, 3]. Additionally, RA patients may have lower muscle mass adjusted for fat mass and lower muscle density, a composite index of intramuscular fat infiltration, compared to healthy individuals [4, 5]. High levels of pro-inflammatory cytokines such as interleukin-1 beta (IL-1β) and tumor necrosis factor-alpha (TNF-α) [6], glucocorticoid treatment [7], exercise intolerance and sedentary behavior [8] are thought to contribute to muscle wasting in RA patients.

Previous studies have reported that low muscle mass is associated with physical disability in RA patients [9–11], and that muscle wasting is associated with increased mortality across several diseases [12], underscoring the clinical importance of screening and monitoring muscle mass. Several methods are available to assess muscle mass, including magnetic resonance imaging (MRI), computed tomography (CT), dual-energy X-ray absorptiometry (DXA) and bioimpedance analysis (BIA) [13–15]. Although these methods demonstrate good validity and agreement, their clinical application is limited by high costs, limited availability, and difficulties in incorporating them into routine practice and longitudinal studies [16].

To address this issue, the use of muscle ultrasound (MU) as a tool for the assessment of muscle mass has emerged as a promising alternative for the improvement of screening and follow-up procedures in clinical practice [14]. MU is used to assess muscle thickness and architecture. It is a relatively fast, safe, and portable method of assessment [17, 18]. In addition, MU measurements have demonstrated good reliability, with strong agreement between repeated assessments and among different examiners [17, 18]. MU can be used to assess five muscle characteristics: muscle thickness, cross-sectional area, fascicle length, pennation angle, and echogenicity [19]. Muscle thickness reflects the amount of muscle mass at a specific anatomical location and is defined as the perpendicular distance between the superficial and deep aponeuroses [20]. This parameter can be used to detect muscle atrophy and hypertrophy [21]. The pennation angle describes the orientation of muscle fibers relative to the tendon and the line of force generation. This architectural feature influences how efficiently muscle force is transmitted [22]. Alterations in muscle thickness and pennation angle are indicative of muscle remodeling and have been associated with disuse [23], aging [24], and chronic inflammatory conditions [16]. Lastly, MU has been shown to be reliable and valid for assessing muscle thickness in older adults when compared to other methods such as MRI, CT and DXA [17].

Previous studies have demonstrated that RA patients have a smaller cross-sectional area of the vastus lateralis (−13.9%) and a smaller pennation angle (−12.5%) compared to healthy age- and sex-matched controls [25]. Blum et al. (2020) [20] also demonstrated that RA women had smaller muscle thickness (−23.3%) and pennation angle (−14.1%) than healthy women. It is known that alterations in muscle morphology by MU can impair autonomy [26] and reduce quality of life [27]. This underscores the importance of monitoring muscle health in RA patients to address these potential challenges.

Although the loss of muscle mass and function has been examined in the existing literature, primarily through cross-sectional studies, the long-term impact of muscle morphological changes on clinical features and health outcomes remains insufficiently investigated. Given the absence of longitudinal studies evaluating muscle morphological changes assessed by ultrasound in RA, the present study adopted an exploratory 1-year follow-up. This timeframe was chosen to examine whether measurable changes in muscle morphology could be identified over a clinically relevant period in routine care. Accordingly, a 12-month observational period was considered appropriate to explore longitudinal muscle changes and their associated predictors. Therefore, the objectives of this study were (1) to evaluate changes in muscle thickness and the pennation angle of the quadriceps muscle assessed by MU over time (1-year), and (2) to identify predictors of changes in muscle morphology over time in RA patients.

## Methods

### Study design

This prospective observational cohort study was conducted at a tertiary public hospital in Rio Grande do Sul, Brazil (Hospital de Clínicas de Porto Alegre, HCPA), including RA patients enrolled between 2018 and 2022 and followed until 2023. Baseline evaluations were performed between 2018 and 2022, with a planned follow-up after one year. However, due to the COVID-19 pandemic, we faced significant disruptions, leading to the loss of follow-up data at the intended one-year time point. Consequently, the follow-up period was extended, with data collected between 2021 and 2023. The institutional review board of the Universidade Federal do Rio Grande do Sul (UFRGS) and Hospital de Clínicas de Porto Alegre (HCPA), Brazil (registered under protocol numbers 2018–0071 and 2021–0550) approved this study. The principles of the Declaration of Helsinki were followed, and all subjects gave written informed consent. This study was reported according to the STROBE Checklist [28]. Clinical trial registration: not applicable.

### Patients

**Fig. 1:**
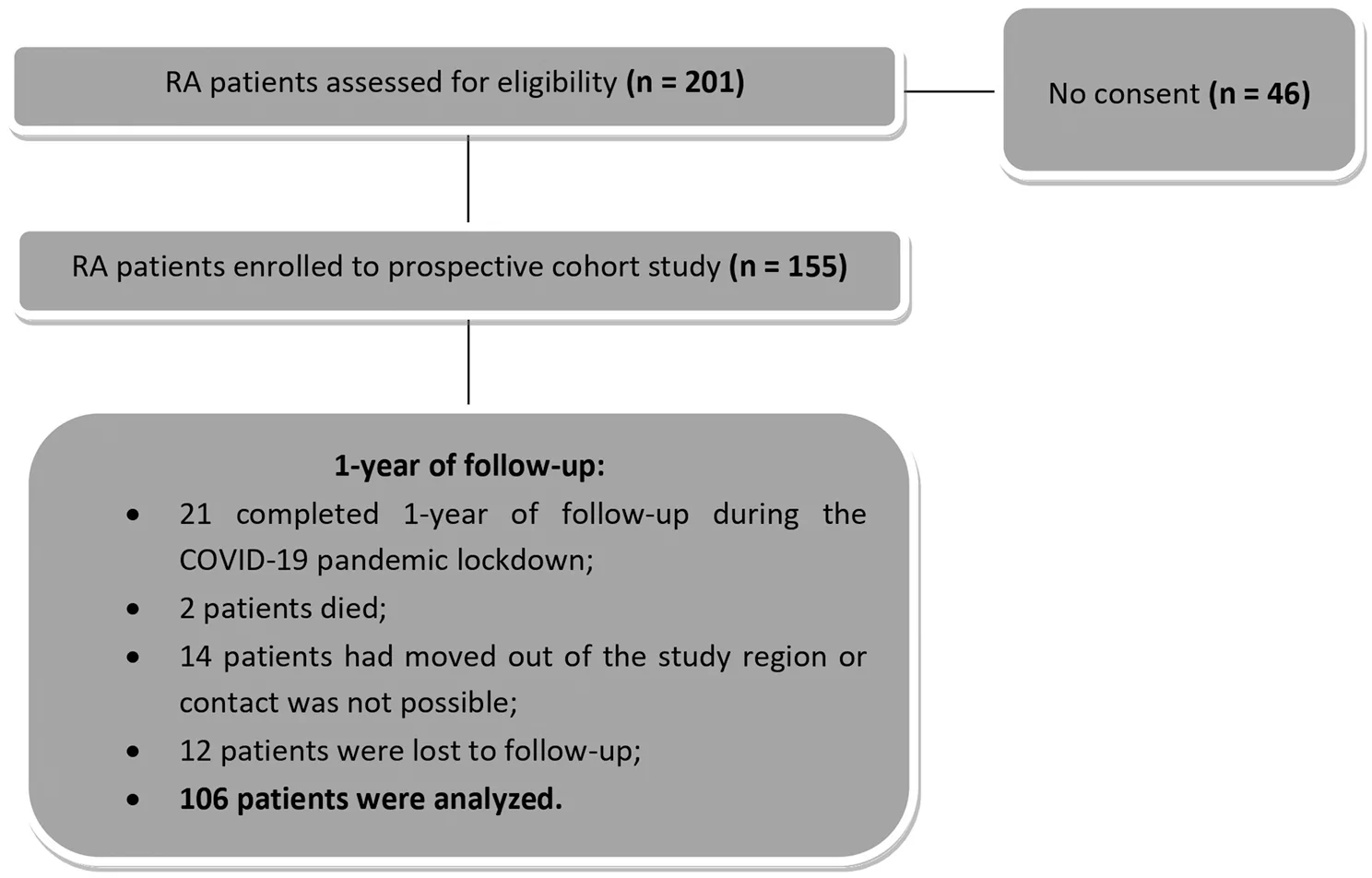
Flow diagram of the study population. Abbreviations: RA, rheumatoid arthritis; n, number

Patients with RA diagnosed according to the 2010 American College of Rheumatology/European League Against Rheumatism (ACR/EULAR) classification criteria [29], aged ≥ 18 years old, and attending the outpatient clinic of the Rheumatology Division at HCPA were consecutively invited to participate. Exclusion criteria comprised malignancy, neuromuscular or metabolic diseases, overlapping autoimmune diseases, inability to walk or flex the knee, a history of knee replacement, and juvenile idiopathic arthritis. Of 201 eligible outpatient patients, 155 RA patients agreed to participate in this prospective study. Of the initial 155 patients, 106 completed the 1-year follow-up (Fig. 1).

### Measurements

Clinical features such as age (years old), disease duration (years), self-reported race/color (white, black or mixed race) defined by the Brazilian Institute of Geography and Statistics (Instituto Brasileiro de Geografia e Estatística- IBGE) [30], bone erosions, current smoking status, rheumatoid factor, anti-citrullinated protein antibodies (anti-CPA), and treatment regimen were assessed through a review of medical records. Disease activity, muscle morphology, muscle strength, physical performance, physical function and physical activity level were recorded by the research team during each study visit.

### Disease activity

Disease activity in RA patients was assessed using the 28-joint Disease Activity Score based on C-reactive protein (DAS28-CRP). The DAS28 includes counts of 28 tender and swollen joints, general health (GH; patient assessment of disease activity using a 100 mm visual analogue scale (VAS), where 0 = best and 100 = worst), and serum levels of C-reactive protein (CRP, mg/L) [31]. Scores < 2.6 indicated remission, scores ≥ 2.6 to <3.2 indicated low disease activity, scores ≥ 3.2 to ≤5.1 indicated moderate disease activity, and scores > 5.1 indicated high disease activity [32].

### Muscle morphology

Morphological parameters of the quadriceps muscle were assessed by MU according to standardized recommendations and consisted of muscle thickness, subcutaneous fat thickness and pennation angle measures [26]. The participants were required to lie supine in a semi-fowler position (hip between 30º–45° and knee semi-flexed) with arms and legs relaxed and in a natural resting position [33]. The images of the rectus femoris, vastus intermedius, and vastus lateralis of the anterior portion were taken at 50% of the thigh length from the greater trochanter to the lateral knee joint space [34, 35]. Each midpoint was clearly marked on the skin with a surgical pen to ensure correct probe placement for repeated scans. The transducer was then lightly placed over the pen mark, perpendicular to the skin and aligned with the limb’s longitudinal axis, at a neutral tilt to obtain a longitudinal ultrasound image of the site. For each measurement, a generous amount of conductive gel was applied to avoid direct contact between the transducer and the skin surface. In addition, the images were acquired under the lightest possible compression to avoid distortion of the muscle tissue. All ultrasound assessments were performed by a single trained and experienced operator (Santos, LP), with experience in muscle ultrasound since 2018, using a real-time ultrasound device (Esaote S.p.A MyLab 50 X Vision; Genoa, Italy) equipped with a 10–18 MHz linear transducer. B-mode images were acquired with the imaging depth typically set at 6 cm and adjusted when necessary to ensure full visualization of the muscle belly. Gain was set at 76% and kept constant across all assessments. The focal zone was positioned at the level of the muscle of interest (focus levels 2–3). Standardized probe positioning and minimal transducer pressure were applied to avoid excessive muscle compression. After proper visualization, the image was captured (“frozen”) and stored on the device’s hard disk as a JPEG file. The examiner repeated this procedure three times at each of the three sites, resulting in nine saved images for each patient [16]. The 1-year follow-up assessment was conducted using the same parameters as the baseline evaluation, following the same standardized procedures. Consistent equipment, anatomical landmarks, room, and patient positioning were used across sessions. Patient positioning was confirmed using a goniometer, and lower-limb mapping was applied to ensure accurate and reproducible probe placement for subsequent assessments.

**Fig. 2:**
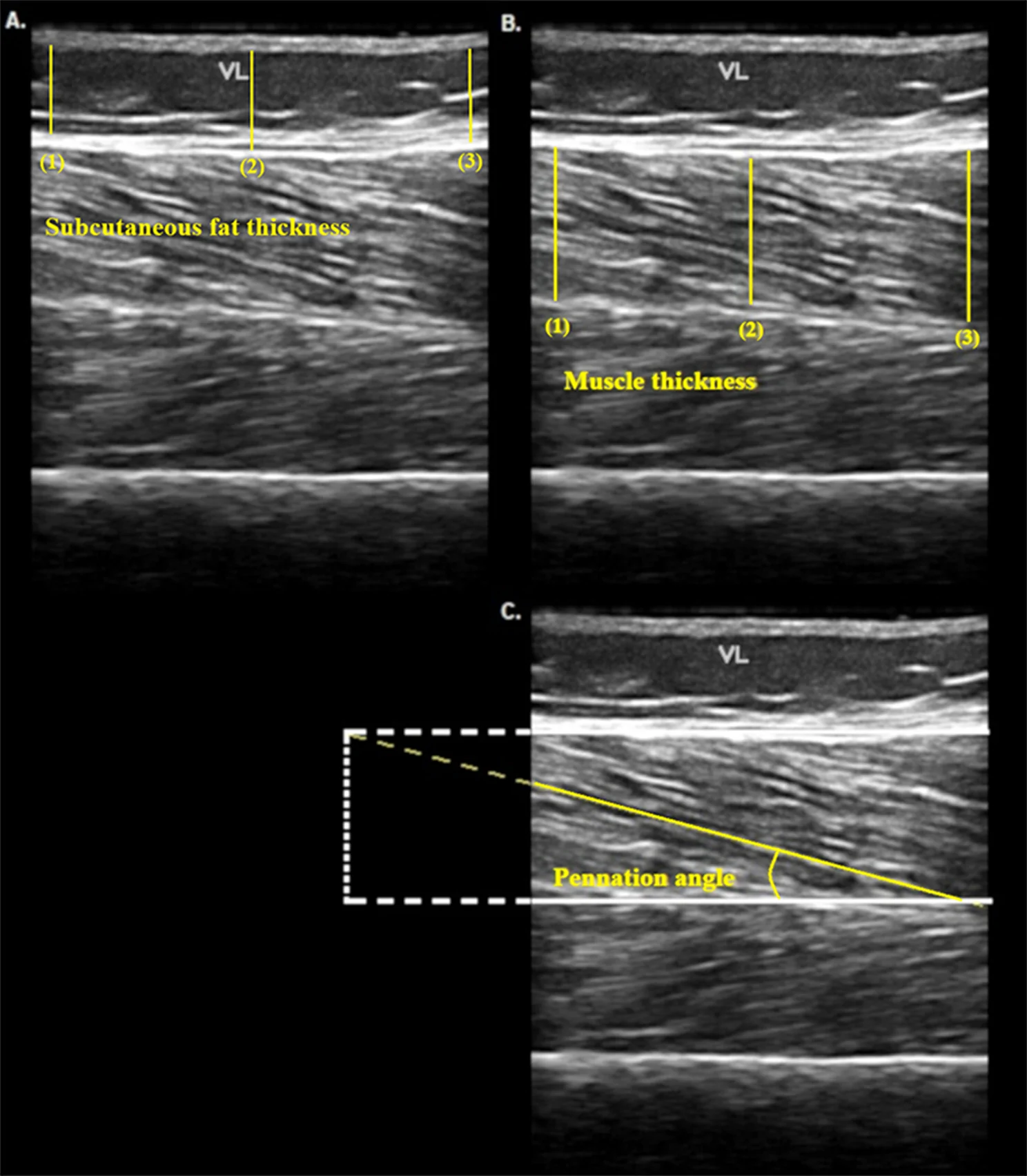
Ultrasound image showing vastus lateralis muscle architecture parameters. (**A**) assessment of subcutaneous fat thickness, (**B**) assessment of muscle thickness and (**C**) assessment of pennation angle. VL: vastus lateralis

The collected images were downloaded for further analysis using the ImageJ program (National Institutes of Health, USA). Before image selection, two evaluators (Santos, LP and Pena, E) jointly screened the image bank to identify and discard images associated with technical issues, such as excessive compression or probe tilt [16]. Thereafter, a single image was selected from each evaluated site (one image per site, totaling three images per patient) [16, 36]. Among the images available at the same location, those with darker muscle tones were preferred as a visual criterion, indicating less compression of the probe [16, 36] and better visualization of the fascicle [16]. If all images collected for a given location did not meet established technical standards that location was considered “absent”, and the patient was excluded from the analysis. To assess muscle thickness, the distance between the deep and superficial aponeuroses was considered and calculated as the mean value of three parallel lines drawn perpendicularly between the superficial and deep aponeuroses along each ultrasound image [16, 20]. Subcutaneous fat thickness was assessed by measuring the distance from the skin to the superficial fascia of the muscle. This measurement was calculated as the mean value of three perpendicular lines drawn along each ultrasound image [37]. For pennation angle analysis, the fascicle with the best visualization within each image was selected. The pennation angle was calculated as the angle between the muscle fascicle and the deep aponeuroses (Fig. 2) [16, 20]. Muscle ultrasound parameters were selected to allow a comprehensive morphological assessment of the quadriceps muscle and its adjacent tissues.

### Muscle strength

Muscle strength was assessed using a handgrip strength test using a handheld dynamometer (Jamar Hydraulic Hand Dynamometer, Preston, USA). The patient was instructed to squeeze the handle as hard as possible for 5 seconds, and the maximal isometric voluntary contraction (MIVC) was quantified. Consistent standardized verbal encouragement was provided throughout the evaluation. The measurement was repeated after a 60-second recovery period, and the highest value from three MIVC attempts in the right hand was considered for analysis. For patients in whom joint involvement impaired testing, specific adjustments to the handheld dynamometer were applied, as described elsewhere [38]. Handgrip strength values < 16 kg (women) and <27 kg (men) were considered indicative of muscle weakness [14, 39].

### Physical performance

Physical performance was assessed by the Short Physical Performance Battery (SPPB). The SPPB is a widely used and simple test that measures lower extremity function through observed completion of tasks that mimic daily actions. The SPPB includes three physical performance domains: balance, walking speed, and the five-time sit-to-stand test. The SPPB score ranges from 0 to 12. A score ≤ 8 points was used to indicate poor physical performance [38, 40, 41].

### Physical disability

Physical disability was assessed by the Health Assessment Questionnaire (HAQ). The HAQ is a questionnaire about the level of difficulty in performing activities of daily living [42]. The HAQ scores were classified as mild (HAQ 0–1), moderate (HAQ 1–2) and severe impairment (HAQ > 2) [43].

### Physical activity levels

Physical activity levels were assessed using the short form of the International Physical Activity Questionnaire (IPAQ) as adapted to Portuguese [44]. Data were collated and reported as median metabolic equivalent of task (MET)-minutes per week, as well as categorized into three activity levels: low, moderate, and high [44]. In addition, during clinical assessments, patients were informally asked about their participation in regular or structured physical exercise (e.g., resistance training, dance, physiotherapy, or other supervised exercise programs). This information was collected to identify potential engagement in muscle strengthening activities during the follow-up period; however, no specific exercise guidance or muscle strengthening instructions were provided by the investigators at any time.

### Statistical analysis

The Shapiro-Wilk method was used to test for normality. Results are expressed as mean ± standard deviation (SD), median (interquartile range, IQR), and number (%), as appropriate. Statistical analysis was performed using paired samples t-tests to assess changes over time. For the modifications in disease activity status, physical function status, physical performance status, and the proportions of drug usage among re-evaluated patients, the McNemar or McNemar-Bowker tests were used when appropriate. Subsequently, Pearson or Spearman correlation coefficients were used, as appropriate, to assess the correlation between clinical features, muscle strength, physical performance, physical disability, and physical activity levels with changes in muscle thickness and pennation angle (Δ, deltas). Correlations were ranked as suggested by Dancey and Reidy [45]: *r* = 1.0 indicates perfect correlation; *r* = 0.7–0.9, strong correlation; *r* = 0.4–0.6, moderate correlation; *r* = 0.1–0.3, weak correlation; and *r* = 0, no correlation. Multiple linear regression analyses were performed to investigate the associations of age, disease duration, disease activity, and handgrip strength (predictive factors) with changes in rectus femoris muscle thickness. These four variables were chosen because they were either correlated with rectus femoris musculature in Pearson’s or Spearman’s analysis or were considered important. Additionally, in multiple linear regression analyzes, multicollinearity was verified by the variance inflation factor (VIF). The acceptable VIF in our study was < 2. The significance level was set at *p* ≤ 0.05 for all analyses. Statistical analyses were performed in the Statistical Package for Social Sciences (SPSS) 17.0.

## Results

Of 201 eligible outpatient clinic patients, 155 RA patients agreed to participate in this prospective observational cohort study. Of the initial 155 patients, 106 completed the 1-year follow-up (Fig. 1). The median follow-up time was 1.3 (interquartile range, IQR: 1.2–1.5) years.

### Clinical features

At baseline, the majority of patients were women (88.4%) (Table 1). The mean ± standard deviation (SD) of age was 58.7 ± 9.5 years old and the median (IQR) of disease duration was 11.0 (6.0–20.0) years. Most patients identified as white (85.2%), with 9.7% identifying as black and 5.2% as mixed race. The mean DAS28-CRP score was 3.0 ± 1.3, and the majority of patients exhibited disease remission (43.5%).

**Table 1:** Clinical features of the RA cohort at baseline

	n	Baseline value
Age (years old), mean ± SD	155	58.7 ± 9.5
Disease duration (years), median (IQR)	155	11.00 (6.00–20.00)
Women, n (%)	155	137 (88.4)
Men, n (%)		18 (11.6)
BMI women, (kg/m^2^), mean ± SD	137	27.90 ± 4.80
BMI men, (kg/m^2^), mean ± SD	18	26.52 ± 5.67
Current smoker, n (%)	155	25 (20.5)
Rheumatoid factor positive, n (%)	155	134 (86.5)
ACPA positive, n (%)	155	90 (58.1)
Presence of erosion, n (%)	155	118 (76.1)
*Disease activity*		
DAS-28–CRP, mean ± SD	154	3.02 ± 1.29
Remission, n (%)		67 (43.5)
Low disease activity, n (%)		24 (15.6)
Moderate disease activity, n (%)		50 (32.5)
High disease activity, n (%)		13 (8.4)
Tender joints, mean ± SD	155	2.66 ± 4.51
Swollen joints, mean ± SD	155	2.42 ± 3.50
VAS, mean ± SD	143	35.97 ± 28.70
CRP (mg/L), median (IQR)	154	5.40 (2.00–10.28)
*Treatment regimen*		
MTX monotherapy*, n (%)	155	62 (40.0)
MTX with concurrent DMARD, n (%)	155	41 (26.5)
MTX dose (mg/week), median (IQR)	103	18.16 ± 4.98
Non-MTX csDMARDs, n (%)	155	49 (31.6)
bDMARDs, n (%)	155	40 (25.8)
tsDMARDs, n (%)	155	11 (7.1)
Glucocorticoids, n (%)	155	43 (27.7)
Glucocorticoid dose (mg/day), median (IQR)	43	5.00 (5.00–10.00)
*Physical disability*		
HAQ, mean ± SD	138	1.09 ± 0.75
Low physical disability, n (%)		71 (51.4)
Moderate physical disability, n (%)		50 (36.2)
High physical disability, n (%)		17 (12.3)
*Physical activity (IPAQ)*	123	
Low, n (%)		47 (38.2)
Moderate, n (%)		32 (26.0)
High, n (%)		44 (35.8)

BMI, body mass index; kg/m^2^, kilogram per square meter; ACPA, anti-citrullinated protein antibodies; DAS-28–CRP, the Disease Activity Score 28 with C-reactive protein; VAS, Visual analogue scale; CRP, C-reactive protein; MTX, Methotrexate; Non-MTX csDMARD, conventional synthetic disease-modifying antirheumatic drugs without MTX (Leflunomide, hydroxychloroquine, and sulfasalazine); bDMARDs, biologic disease-modifying antirheumatic drugs (adalimumab, etanercept, abatacept, infliximab, certolizumab, golimumab, rituximab, tocilizumab); tsDMARDS, target synthetic disease-modifying antirheumatic drugs (Tofacitinib, baracitinib); HAQ, Health Assessment Questionnaire; IPAQ, International Physical Activity Questionnaire; n, number; SD, standard deviation; IQR, Interquartile; %, percent; mg, milligram;*MTX with or without glucocorticoids

The mean physical function score, assessed by the HAQ, was 1.09 ± 0.75. Among the patients, 48.5% had a moderate to severe impairment of physical function. According to the IPAQ, 38.2% of patients had low physical activity levels, 26.0% had moderate levels, and 35.8% had high levels. In regarding to treatment regime, 40.0% were on methotrexate (MTX) monotherapy, 26.5% were receiving MTX in combination with other disease-modifying antirheumatic drugs (DMARDs), 25.8% were using biologic disease-modifying antirheumatic drugs (bDMARDs), 7.1% were using targeted synthetic disease-modifying antirheumatic drugs (tsDMARDs), and 27.7% were using glucocorticoids. Additional demographic and clinical details are described in Table 1.

After one year of follow-up, the mean disease activity measured by DAS28-CRP, tender joints, visual analog scale (VAS), CRP, and physical function of the re-evaluated patients did not change (*p* > 0.05). The number of swollen joints decreased by 38.3% (*p* = 0.003). Additionally, women experienced a decrease of 1.4% in body mass index (BMI) over time (*p* = 0.028). The details are shown in Table 2.

**Table 2:** Clinical features of RA cohort study

	n	At baseline	n	At the end follow-up	p
Women, n (%)	106	96 (90.6)	106	96 (90.6)	
Men, n (%)		10 (9.4)		10 (9.4)	
BMI women, (kg/m^2^), mean ± SD	96	27.34 ± 4.86	96	26.95 ± 4.50	**0.028**
BMI men, (kg/m^2^), mean ± SD	10	27.76 ± 5.96	10	28.02 ± 5.78	0.747
*Disease activity*					
DAS-28–CRP, mean ± SD	105	3.02 ± 1.23	105	2.94 ± 1.17	0.516
Tender joints, mean ± SD	106	2.43 ± 4.09	106	2.48 ± 3.84	0.923
Swollen joints, mean ± SD	106	2.35 ± 3.02	106	1.45 ± 2.46	**0.003**
VAS, mean ± SD	97	38.95 ± 28.50	97	35.11 ± 30.07	0.291
CRP (mg/L), median (IQR)	105	5.00 (1.95–9.40)	105	4.50 (1.50–11.85)	0.848
*Physical disability*					
HAQ-DI, mean ± SD	92	1.09 ± 0.70	92	1.03 ± 0.76	0.248
*Physical activity levels*					
IPAQ (MET-min.), median (IQR)	86	676.00 (0.00–3459.75)	86	1339.50 (480.00–3819.00)	0.117
Women’s IPAQ (MET-min.), median (IQR)	76	694.00 (0.00–3456.75)	76	1339.50 (541.88–3360.00)	0.064
Men’s IPAQ (MET-min.), median (IQR)	10	259.00 (74.25– 14,983.50)	10	2108.25 (247.50–6777.50)	0.799

BMI, body mass index; kg/m^2^, kilogram per square meter; DAS-28–CRP, the Disease Activity Score 28 with C-reactive protein; VAS, Visual analogue scale; CRP, C-reactive protein; HAQ, Health Assessment Questionnaire; IPAQ, International Physical Activity Questionnaire; MET-min., Metabolic Equivalent of Task – minutes; n, number; SD, standard deviation; IQR, Interquartile; %, percent

Additionally, disease activity status (remission, low, moderate, and high disease activity), physical disability status (mild, moderate, and severe impairment), physical performance (poor and good physical performance), and physical activity status (low, moderate, and high physical activity) did not change (*p* > 0.05). Furthermore, no changes related to physical exercise were observed (*p* > 0.05). More details are shown in supplementary Figure 1 and supplementary Table 1. Similarly, the proportion of patients using MTX (*p* = 0.210), bDMARDs (*p* = 0.302) and use of glucocorticoids (*p* = 0.115) did not change. Of the six re-evaluated patients who were using tsDMARDs, all maintained their usage. Additionally, seven patients started using the drug, resulting in a 7% increase in tsDMARDs usage (*p* = 0.016). Regarding non-MTX csDMARDs (Leflunomide, hydroxychloroquine, and sulfasalazine), among the 39 re-evaluated patients, 3 discontinued use and 16 initiated non-MTX csDMARDs therapy during follow-up, resulting in an overall 15% increase in non-MTX csDMARDs use (*p* = 0.004). More details are shown in supplementary Figure 2 and supplementary Table 2.

### Muscle morphology

**Fig. 3:**
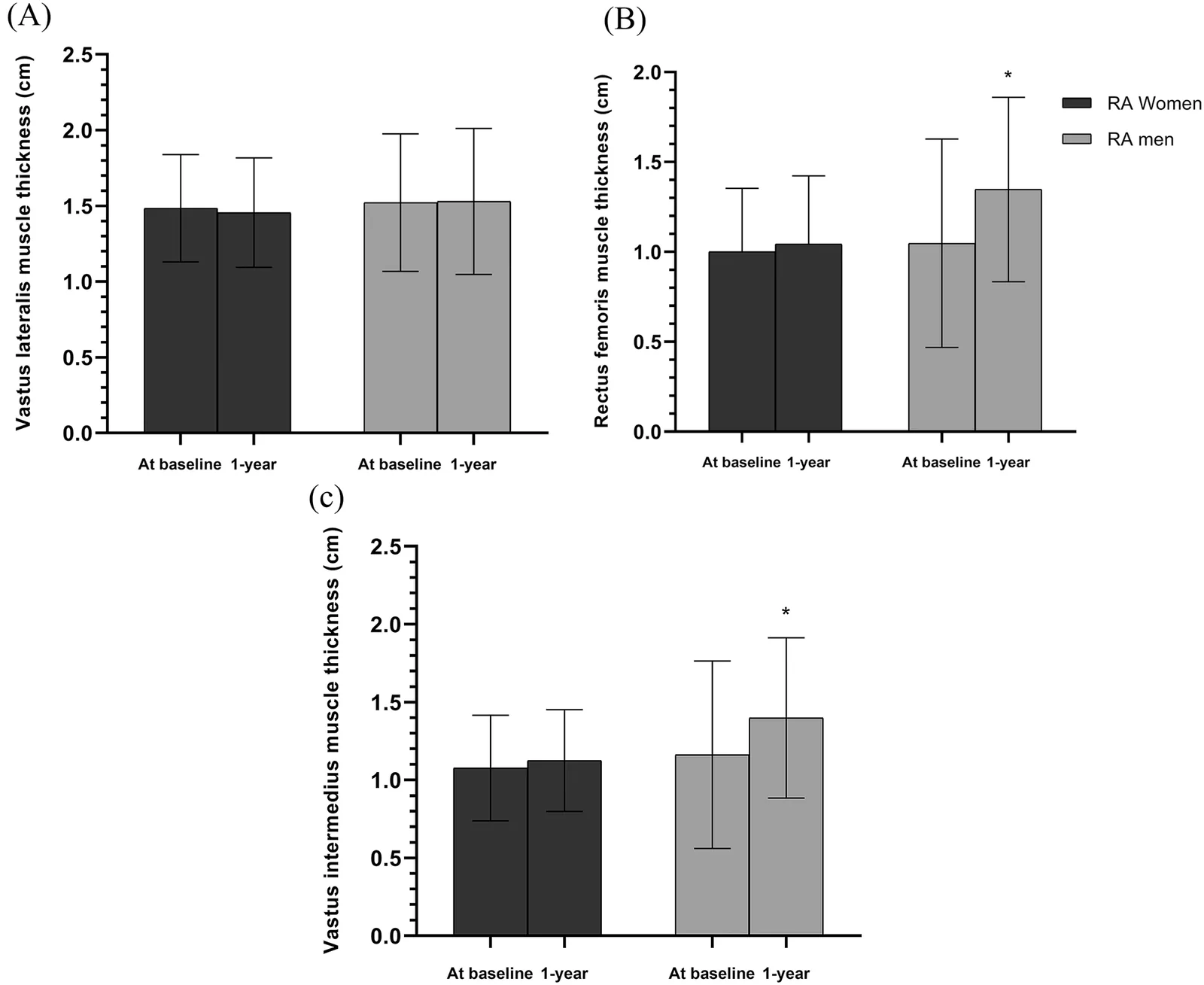
Changes in muscle thickness of women and men after 1-year. (**A**): vastus lateralis muscle thickness; (**B**): rectus femoris muscle thickness (**C**): vastus intermedius muscle thickness. Abbreviations: cm, centimeters; RA, rheumatoid arthritis; * statistical difference between at baseline and 1-year

**Fig. 4:**
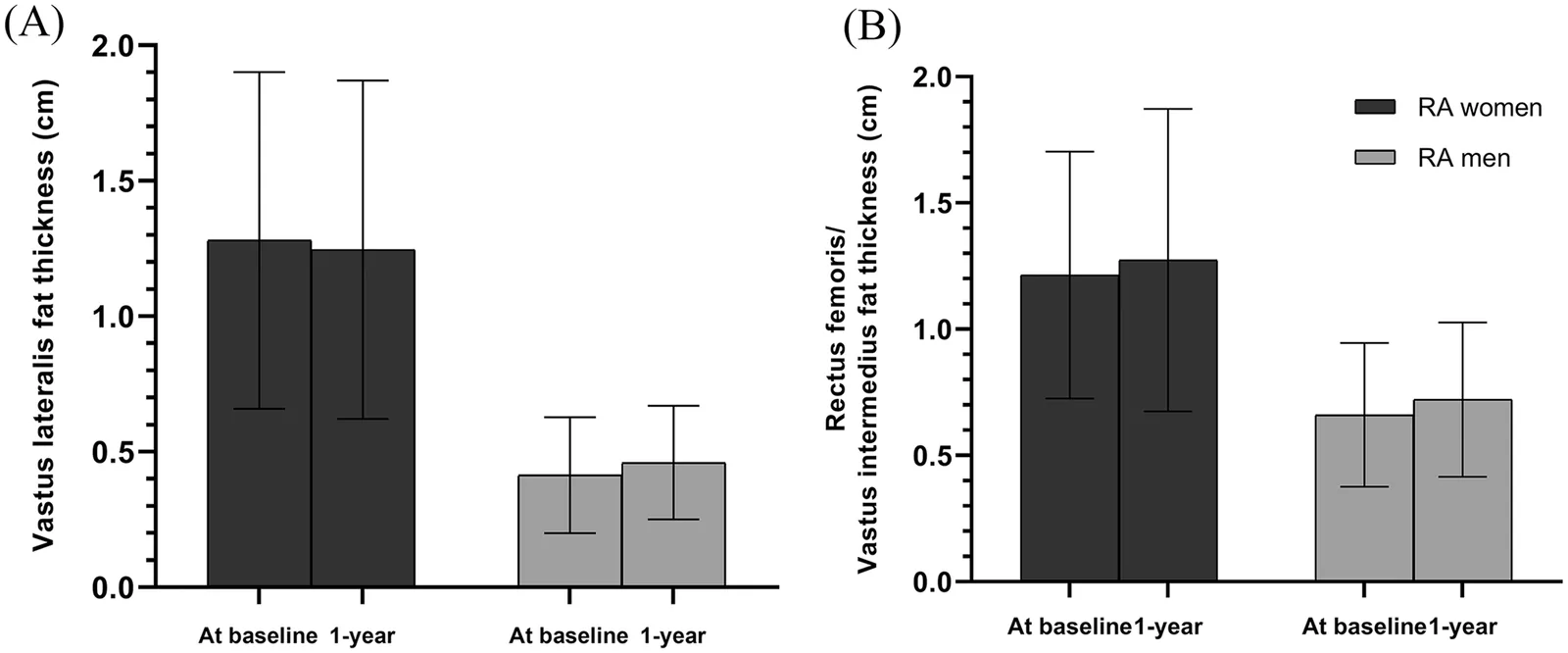
Changes in fat thickness of women and men after 1-year. (**A**): fat thickness of the vastus lateralis; (**B**): fat thickness of rectus femoris and vastus intermedius. Abbreviations: cm, centimeters; RA, rheumatoid arthritis; * statistical difference between at baseline and 1-year

**Fig. 5:**
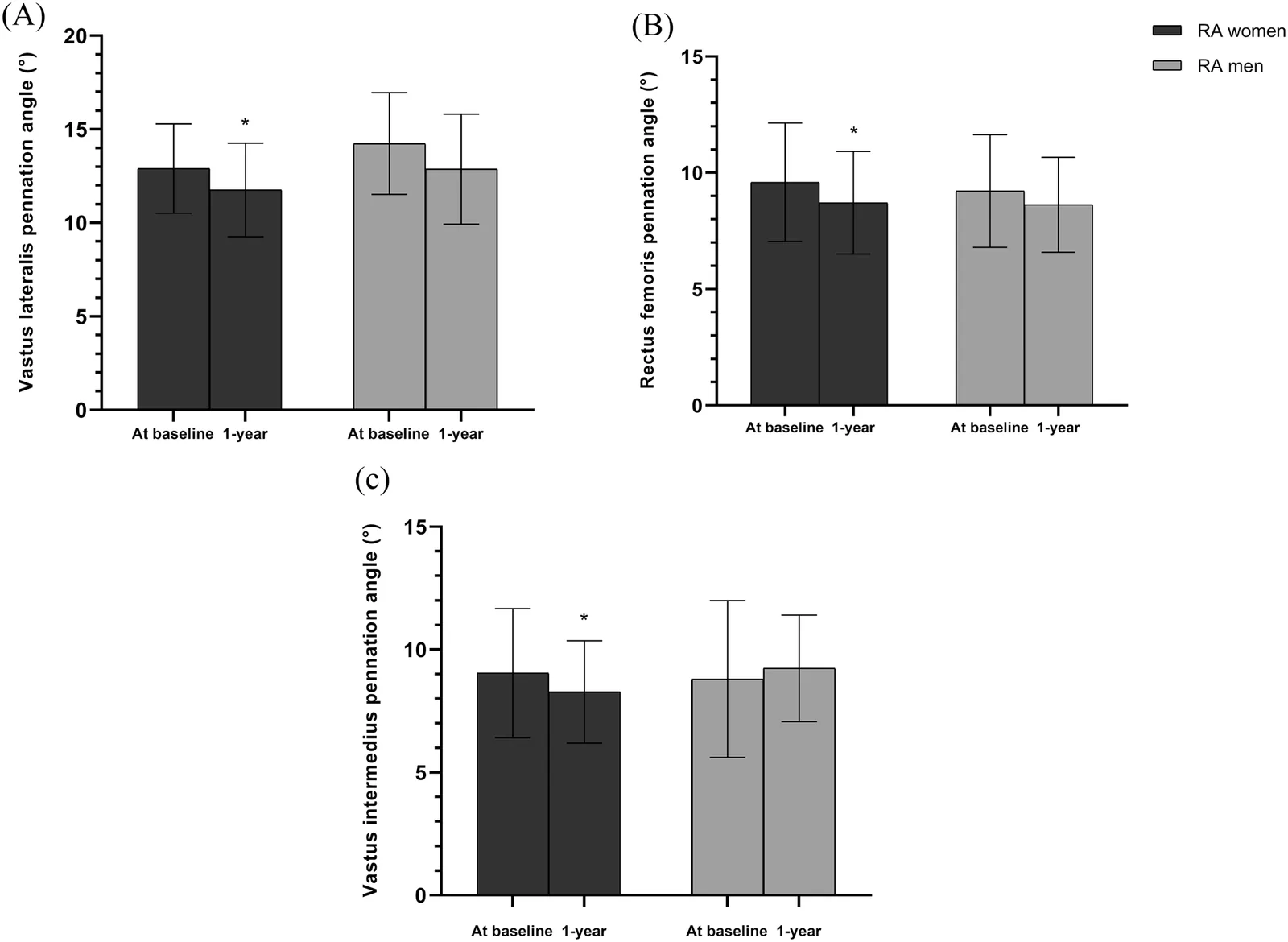
Changes in pennation angle of women and men after 1 year. (**A**): vastus lateralis muscle thickness; (**B**): rectus femoris muscle thickness (**C**): vastus intermedius muscle thickness. Abbreviations: °, degrees; RA, rheumatoid arthritis; * statistical difference between at baseline and 1-year

After 1-year of follow-up, the women (*n* = 96) did not show any significant changes in muscle thickness (*p* > 0.05, Fig. 3) or subcutaneous fat thickness (*p* > 0.05, Fig. 4). However, they exhibited a significant decrease in the pennation angle: 7% in the vastus lateralis (*p* < 0.001), 6% in the rectus femoris (*p* < 0.001), and 4% in the vastus intermedius (*p* = 0.002) (Fig. 5).

The re-evaluated men (*n* = 10) showed a 39% increase in rectus femoris muscle thickness (*p* = 0.010) and a 29% increase in vastus intermedius muscle thickness (*p* = 0.019) (Fig. 3). No significant changes were observed in vastus lateralis muscle thickness (Fig. 3), subcutaneous fat thickness (Fig. 4), or the pennation angle (*p* > 0.05, Fig. 5).

### Changes in muscle strength and physical performance

**Fig. 6:**
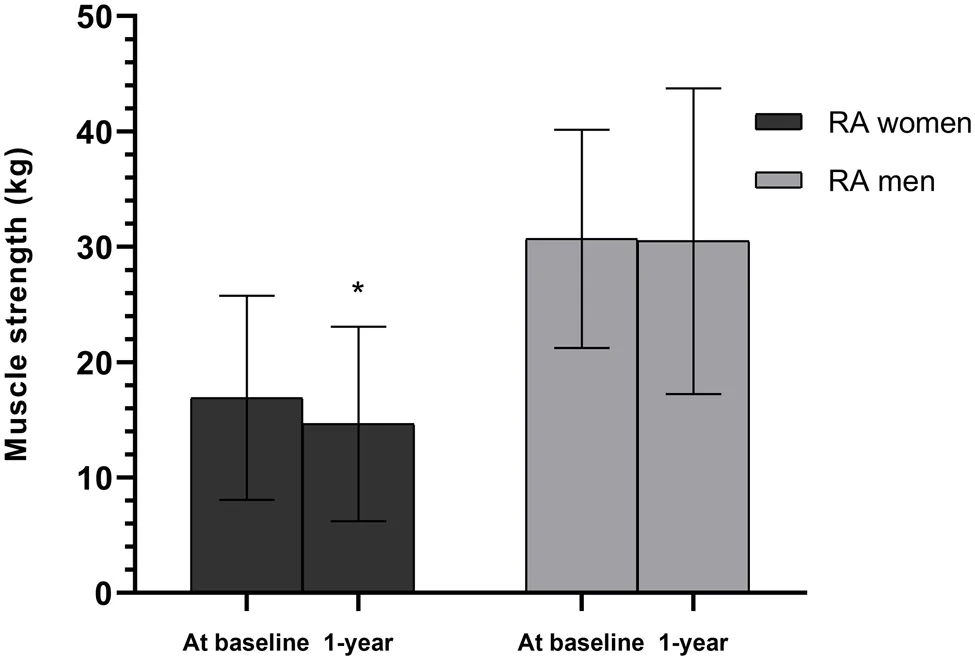
Changes in muscle strength of women and men after 1-year. RA, rheumatoid arthritis; kg, kilogram; * statistical difference between at baseline and 1-year

At the 1-year, women patients re-evaluated (*n* = 95) showed a decrease in the muscle strength of 4% (*p* = 0.001; Fig. 6). On the other hand, no changes in physical performance in women were found. Additionally, muscle strength and physical performance did not change in men (*p* > 0.05). The frequency of low muscle strength and low physical performance did not change (*p* > 0.05).

### Correlated and predictors factors of changes in muscle morphology over time

**Fig. 7:**
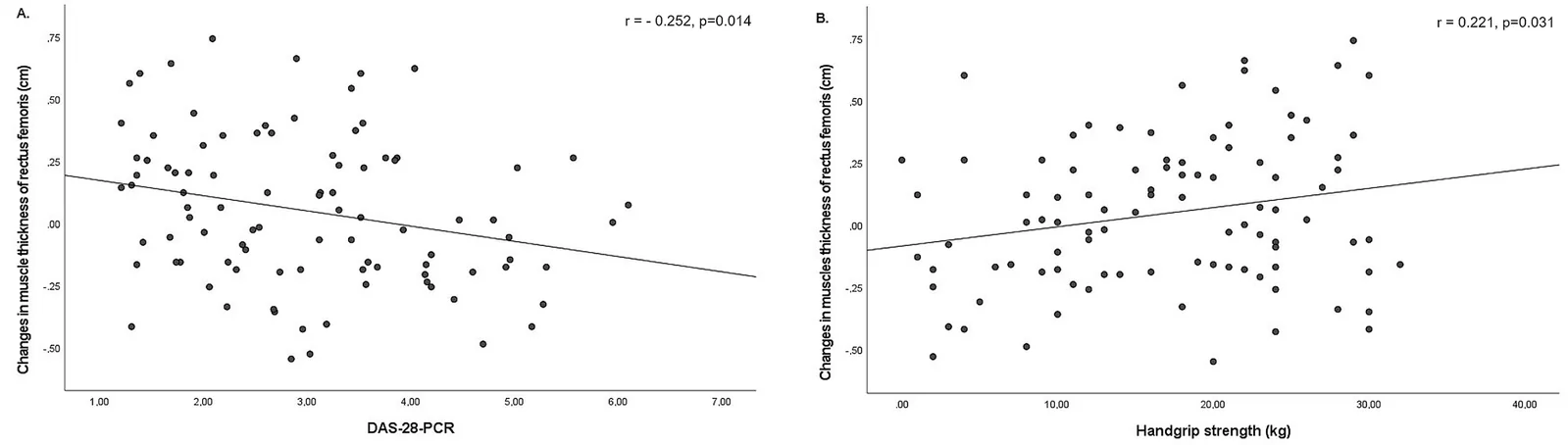
(**A**). Correlation of disease activity by DAS28-CRR with changes in muscle thickness of rectus femoris; (**B**). Correlation of handgrip strength with changes in muscle thickness of rectus femoris

Due to the limited number of men in our sample (*n* = 10), correlation analyses were conducted exclusively on the re-evaluated women. A lower DAS28-CRP at baseline was correlated with larger increases in rectus femoris muscle thickness (*r* = − 0.252, *p* = 0.014) at follow-up. Moreover, greater handgrip muscle strength at baseline was correlated with larger increases in rectus femoris muscle thickness (*r* = 0.221, *p* = 0.031) in follow-up. The data are shown in Fig. 7.

There was an association between DAS–28 and rectus femoris thickness. For each unit increase in DAS-28-CRP, the rectus femoris thickness decreased by 0.058 cm (β = −0.058; CI 95%, −0.114 to −0.003; *p* = 0.039). Although not statistically significant, there was a trend toward a negative association between age and changes in rectus femoris muscle thickness (β = −0.004; 95% CI, −0.011 to 0.003). In contrast, muscle strength showed a trend toward a positive association with changes in rectus femoris muscle thickness (β = 0.004; CI 95%, −0.004 to 0.012). Lastly, total disease duration (β = −0.002; CI 95%, −0.009 to 0.005) had a very small, non-significant negative effect on muscle thickness.

Lastly, patients who used glucocorticoids at baseline (*n* = 30) exhibited a decrease of 0.027 cm in rectus femoris muscle thickness, while patients who did not use glucocorticoids (*n* = 76) exhibited an increase of 0.10 cm (*p* = 0.045). However, the median baseline glucocorticoid dosage (5.00 [5.00–11.25] mg/day) was not associated with changes in muscle thickness, fat thickness and pennation angle.

### Comparison of evaluated versus non-evaluated rheumatoid arthritis patients

Comparing the baseline data of patients who were re-evaluated with those who were not, we observed that women who discontinued the reassessment had higher BMI, greater rectus femoris thickness, and increased vastus intermedius (*p* < 0.05). No other significant differences were found between re-evaluated patients and those who discontinued. More details are described in Table 3.

**Table 3:** Clinical difference between re-evaluated and not re-evaluated patients

Variable	n	Re-evaluated	n	Not re-evaluated	p
Age (years old), mean ± SD	106	59.36 ± 8.96	49	57.16 ± 10.48	0.181
Disease duration (years), median (IQR)	106	12.00 (7.75–22.00)	49	9.00 (3.00–13.50)	0.122
BMI, (kg/m^2^), mean ± SD					
Women	96	27.34 ± 4.85	41	29.23 ± 4.43	**0.033**
Men	10	27.76 ± 5.96	8	24.96 ± 5.25	0.313
DAS-28–CRP, mean ± SD	105	3.02 ± 1.23	49	3.03 ± 1.42	0.970
CRP, median (IQR)	105	5.00 (1.95–9.40)	49	6.70 (2.30–10.75)	0.305
Tender joints, mean ± SD	106	2.43 ± 4.09	49	3.14 ± 5.32	0.365
Swollen joints, mean ± SD	106	2.35 ± 3.02	49	2.57 ± 4.39	0.714
VAS, mean ± SD	98	38.99 ± 28.36	45	29.38 ± 28.67	0.063
HAQ, mean ± SD	92	1.09 ± 0.70	46	1.09 ± 0.84	0.981
IPAQ, median (IQR)	86	676.00 (0.00–3459.75)	37	792.00 (190.50–4339.00)	0.440
Rectus femoris muscle thickness (cm), mean ± SD					
Women	96	1.00 ± 0.35	41	1.20 ± 0.40	**0.004**
Men	10	1.05 ± 0.58	8	1.19 ± 0.42	0.566
Vastus intermedius muscle thickness (cm), mean ± SD					
Women	96	1.08 ± 0.34	41	1.20 ± 0.37	**0.050**
Men	10	1.16 ± 0.60	8	1.12 ± 0.39	0.862
Vastus lateralis muscle thickness (cm), mean ± SD					
Women	96	1.48 ± 0.35	41	1.61 ± 0.44	0.081
Men	10	1.52 ± 0.45	8	1.61 ± 0.44	0.698
Subcutaneous fat thickness of the rectus femoris/vastus intermedius (cm), mean ± SD					
Women	96	1.21 ± 0.49	41	1.28 ± 0.48	0.473
Men	10	0.66 ± 0.28	8	0.56 ± 0.21	0.439
Subcutaneous fat thickness of the vastus lateralis (cm), mean ± SD					
Women	96	1.28 ± 0.62	41	1.24 ± 0.48	0.717
Men	10	0.41 ± 0.21	8	0.35 ± 0.23	0.554
Pennation angles of the rectus femoris (°), mean ± SD					
Women	96	9.60 ± 2.55	41	9.68 ± 2.86	0.872
Men	10	9.22 ± 2.42	8	10.45 ± 1.23	0.212
Pennation angles of the vastus intermedius (°), mean ± SD					
Women	96	9.04 ± 2.63	41	9.21 ± 2.89	0.737
Men	10	8.80 ± 3.19	8	9.48 ± 1.23	0.546
Pennation angles of the vastus lateralis (°), mean ± SD					
Women	96	12.91 ± 2.39	41	12.41 ± 2.66	0.280
Men	10	14.24 ± 2.72	8	12.40 ± 2.68	0.169
Handgrip (kg), mean ± SD					
Women	95	16.92 ± 8.54	41	15.15 ± 9.67	0.289
Men	10	30.70 ± 9.46	8	22.38 ± 12.37	0.125

BMI, body mass index; kg/m^2^, kilogram per square meter; DAS-28–CRP, the Disease Activity Score 28 with C-reactive protein; VAS, Visual analogue scale; CRP, C-reactive protein; HAQ, Health Assessment Questionnaire; IPAQ, International Physical Activity Questionnaire; n, number; SD, standard deviation; IQR, Interquartile; Women (*n* = 96 vs. 41); Men (*n* = 10 vs. 8)

## Discussion

Our prospective study is an original study that used ultrasound to assess muscle thickness, fat thickness, and pennation angle, providing valuable insights into changes in muscle thickness and pennation angle in RA patients over a one-year follow-up period. We observed a decrease in the pennation angles of the vastus lateralis, rectus femoris, and vastus intermedius muscles in women with RA, despite stable or reduced disease activity and consistent drug usage patterns. Additionally, lower baseline disease activity, assessed by the DAS28-CRP, and higher baseline muscle strength, assessed by the handgrip test, were correlated with increased muscle thickness at the end of follow-up in RA women. Among these, DAS28-CRP emerged as a significant predictor of muscle thickness changes, explaining a substantial portion of the variation observed. Furthermore, glucocorticoid use at baseline corresponded with a reduction in rectus femoris muscle thickness at the end of follow-up. On the other hand, RA men exhibited an increase in rectus femoris and vastus intermedius muscle thickness, however, the sample size was too limited to make any interpretation.

The MU has emerged as a widely accepted and extensively researched tool for assessing muscle thickness and architecture [17, 18]. Currently, MU is gaining prominence in the screening and diagnosis of sarcopenia [46]. Cross-sectional studies in RA have highlighted its utility. In a previous study conducted by our research group [20], 35 RA women were compared with 35 healthy controls, demonstrating that RA patients exhibited 23.3% lower vastus lateralis muscle thickness and a 14.1% lower pennation angle compared to healthy controls. However, no significant associations were observed between muscle thickness or pennation angle and clinical features, physical function, muscle strength, pain, and glucocorticoid use [20]. Similarly, Matschke et al. (2010) [25] reported a 13.9% smaller cross-sectional area and a 12.5% lower pennation angle of the vastus lateralis in RA patients. Despite these changes in muscle morphology, specific muscle force was preserved, and changes in muscle morphology did not significantly impact muscle function [25]. The differences in muscle thickness and pennation angle of RA patients compared to healthy controls, as reported in the literature [20, 25], raise concerns regarding the potential presence of sarcopenia in this population.

In this context, Tada et al. (2021) [47] assessed 84 RA patients to verify the usefulness of MU in screening for sarcopenia and obesity. The current study identified sarcopenia in 22.6% of the enrolled patients, in accordance with the Asian Working Group for Sarcopenia criteria. Furthermore, the prevalence was 44.4% among men and 16.7% among women. The authors proposed a cut-off for sarcopenia diagnosis based on combined muscle thickness of the rectus femoris and vastus intermedius: ≤19.7 mm in women and ≤24.7 mm in men. The cut-off for diagnosing obesity was a fat thickness of ≥14.6 mm in women and ≥8.1 mm in men [47]. Using this cut-off, our study observed 53 women (38.7%) and 12 men (66.7%) with sarcopenia (data not published). The mean age and disease activity in our population were lower than those observed in the Tada et al. (2021) study [47]. While muscle strength in women was comparable, the men in our cohort demonstrated greater muscle strength. However, the evaluation positions differed between studies, which may potentially influence these measurements [48].

There are no longitudinal studies that have used muscle ultrasound to monitor changes in muscle morphology over time in RA. However, muscle ultrasound has been employed in other populations, such as hospitalized older adults, to assess muscle morphology changes. Nagae et al. (2022) [49] assessed 145 hospitalized older adults and found minimal decreases in the bilateral anterior thigh muscle thickness over a week, suggesting muscle loss during acute hospitalization periods. In another study, Meza-Valderrama et al. (2022) [50] observed increases in rectus femoris muscle thickness and an increase in cross-sectional area over a 2-week period in a post-acute care hospitalization. Patients underwent an individualized rehabilitation program according to their functional needs, highlighting the importance of rehabilitation in preserving muscle morphology. Muscle ultrasound is a valuable tool due to its capacity to provide reliable, real-time measurements of muscle structure, thus making it particularly suitable for monitoring subtle changes in muscle mass over time. The non-invasive nature, portability, and ease of use of this diagnostic tool offer distinct advantages in clinical practice, especially in settings where more expensive imaging techniques may not be accessible. Although its longitudinal application in RA remains unexplored, its use in other populations suggests that it could be an important tool for tracking muscle changes and guiding early interventions. Furthermore, Paramalingam et al. (2024) [51] followed patients with idiopathic inflammatory myopathy for up to four visits, with intervals of 3–6 months between each visit. An increase in echogenicity was observed in the VL muscle in the prevalent disease group, and interesting trends were noted, showing a reduction in echogenicity, which returned to normal in the incident group after treatment. This study highlights the potential of ultrasound for longitudinal monitoring, similar to what could be explored in the context of RA, where subtle changes in muscle morphology could be tracked using this tool.

Our study found a decrease in vastus lateralis, rectus femoris and vastus intermedius pennation angles, as well as a decrease in muscle strength in women re-evaluated at 1-year. Conversely, the men re-evaluated exhibited an increase in rectus femoris and vastus intermedius muscle thickness at 1-year. Although longitudinal data on changes in muscle morphology in RA are limited, our findings underscore the utility of MU in monitoring muscle health over time in these patients. Considering that MU has demonstrated high reliability and validity in assessing muscle thickness, particularly in large muscle groups such as the quadriceps, and shows strong correlation with methods like DXA, MRI, and CT [17], it represents a valuable, non-invasive tool for tracking muscle changes in RA patients. RA patients often exhibit reduced muscle thickness and pennation angle compared to healthy controls [20, 25]. Additionally, the aging process is associated with an approximately 13% reduction in pennation angles compared to younger individuals [52]. The decrease in pennation angles observed in RA women highlights the importance of monitoring muscle changes and disease progression, as this tool allows for rapid, non-invasive detection of subtle alterations in muscle structure that may be associated with disease activity or muscle strength. In contrast, the improvement in muscle thickness observed in men may be related to higher physical activity levels, as indicated by their higher self-reported IPAQ scores at the end of the follow-up. This suggests that physical activity might positively impact muscle morphology. However, the small sample size of men in our study should be considered when interpreting these results.

When verifying the predictive factors, we found that both disease activity, assessed by DAS28-CRP, and muscle strength were correlated with changes in rectus femoris muscle thickness, although only DAS28-CRP was a significant predictor of these changes. It is well established that disease activity is associated with elevated levels of circulating inflammatory markers, which in turn play an important role in the reduction of muscle mass and skeletal muscle strength [53]. Furthermore, prolonged duration of the disease is associated with increased inflammatory states and a history of steroid use in some cases, as a recognized risk factor for sarcopenia, which is characterized by low muscle strength and low muscle mass [54, 55]. Our patients had established disease with a median of 11.00 (6.00–20.00) years of disease duration, which may represent a high cumulative inflammatory load. In addition, patients who were on glucocorticoids at baseline exhibited a decrease in rectus femoris muscle thickness, whereas those who did not use glucocorticoids showed an increase. Yamada et al. (2020) [7] reported that an average glucocorticoid dose ≥ 3.25 mg/day over one year significantly increased the risk of sarcopenia in patients with RA. While our study did not find a direct association between the baseline glucocorticoid dose and changes in muscle morphology, it is important to note that our measurement reflects the dose at a single time point rather than the cumulative dose. Assessing the cumulative dose would offer a more comprehensive insight into the long-term effects on muscle health.

This study has some limitations. Firstly, we were able to assess 68% of our baseline sample. There were some differences between patients who were re-evaluated and those who were not re-evaluated. In particular, women who were not reassessed exhibited a higher BMI and greater rectus femoris and vastus intermedius muscle thickness. No other significant differences were observed between the re-evaluated patients and those who were not re-evaluated. It is important to note that 21 patients dropped out during the lockdown due to the COVID-19 pandemic, and two patients died. However, the sample size was sufficient to demonstrate significant differences in the muscle morphology characteristics. Additionally, a control group of age- and sex-matched healthy women and men was not included for comparative analysis. Furthermore, the number of men recruited and analyzed was low, leading to their exclusion from some analyses. Consequently, it would be beneficial to evaluate more RA men to better understand changes in their body composition and clinical parameters over time. Moreover, the nutritional status of the patients was not assessed. Additionally, muscle strength was assessed using handgrip dynamometry, which does not directly correspond to the quadriceps muscle evaluated by ultrasound; therefore, a direct local structure–function relationship could not be established. Handgrip strength was included as a feasible and widely accepted marker of global muscle strength in RA. Importantly, recent evidence demonstrates significant associations between quadriceps muscle morphology assessed by ultrasound and both handgrip strength (*r* = 0.70, *p* < 0.001) and knee extension strength (*r* = 0.69, *p* < 0.01) [56], supporting the interpretation of quadriceps ultrasound as a marker of global muscle status. Nevertheless, future studies should incorporate quadriceps-specific strength assessments, such as knee extension dynamometry, to better elucidate direct structure–function relationships. Lastly, no a priori sample size or power calculation was performed, as no longitudinal ultrasound studies in RA were available to inform effect size estimates at the time of study design. Consequently, the results should be interpreted with caution.

Despite these limitations, this study presents several strengths. We conducted a comprehensive 1-year prospective observational cohort study, providing valuable longitudinal data on changes in muscle morphology in RA patients. These findings highlight significant changes in muscle morphology and muscle strength, particularly in RA women, and offer new insights into the impact of disease activity and glucocorticoids use on muscle health. To our knowledge, this is the first study to evaluate these changes in quadriceps muscle morphological parameters by ultrasound over time in this patient population, highlighting the importance of our contributions to the understanding of muscle changes related to RA.

## Conclusion

In this 1-year prospective observational cohort, RA women demonstrated reductions in quadriceps muscle morphology and muscle strength over time. Baseline disease activity emerged as an important predictor of these longitudinal changes. In addition, patients using glucocorticoids at baseline showed less favorable muscle thickness changes compared with non-users, although glucocorticoid dose was not associated with muscle outcomes.

These findings suggest that disease activity and glucocorticoid exposure may contribute to muscle morphological changes in RA. As such, muscle ultrasound proved to be a feasible and practical tool for the longitudinal assessment of muscle morphology in a routine outpatient setting. Future studies with larger samples, the inclusion of healthy controls, detailed assessment of cumulative glucocorticoid exposure, nutritional status, and greater representation of men are warranted to further clarify the determinants of muscle changes in RA.

## Electronic supplementary material

Below is the link to the electronic supplementary material.

Supplementary Material 1

Supplementary Material 2

Supplementary Material 3

Supplementary Material 4

## Data Availability

All data supporting the findings of this study are available within the paper and its Supplementary Information.

## Abbreviations

ACR/EULAR: American College of Rheumatology/European League Against Rheumatism
anti-CPA: Anti-citrullinated protein antibodies
bDMARDs: Biologic disease-modifying antirheumatic drugs
BIA: Bioimpedance
BMI: Body mass index
CI: Confidential interval
CRP: C-reactive protein
CT: Computed tomography
DAS–28: 28-joint Disease activity
DMARDs: Disease-modifying antirheumatic drugs
DXA: Dual-energy X-ray absorptiometry
HAQ: Health Assessment Questionnaire
HCPA: Hospital de Clínicas de Porto Alegre
IBGE: Brazilian Institute of Geography and Statistics
IL-1β: Interleukin-1 beta
IPAQ: International Physical Activity Questionnaire
IQR: Interquartile range
MET: Metabolic equivalent of task
MICV: Maximal isometric voluntary contraction
MRI: Magnetic resonance imaging
MTX: Methotrexate
RA: Rheumatoid arthritis
RF: Rectus femoris
SD: Standard deviation
SPSS: Statistical Package for Social Sciences
TNF-α: Tumor necrosis factor-alpha
tsDMARDs: Targeted synthetic disease-modifying antirheumatic drugs
UFRGS: Universidade Federal do Rio Grande do Sul
MU: Muscle ultrasound
VAS: Visual analog scale
VI: Vastus intermedius
VIF: Variance inflation factor
VL: Vastus lateralis
