# Supplementary material for: Muscle morphology changes over 1-year in patients with rheumatoid arthritis: a prospective cohort study using muscle ultrasound
Source: Adv Rheumatol. 2026 Jul 21;66:46. doi: 10.1186/s42358-026-00529-z (PMC13488837; doi:10.1186/s42358-026-00529-z)
Supplement: Supplementary file 1 [file 42358_2026_529_MOESM1_ESM.pdf]

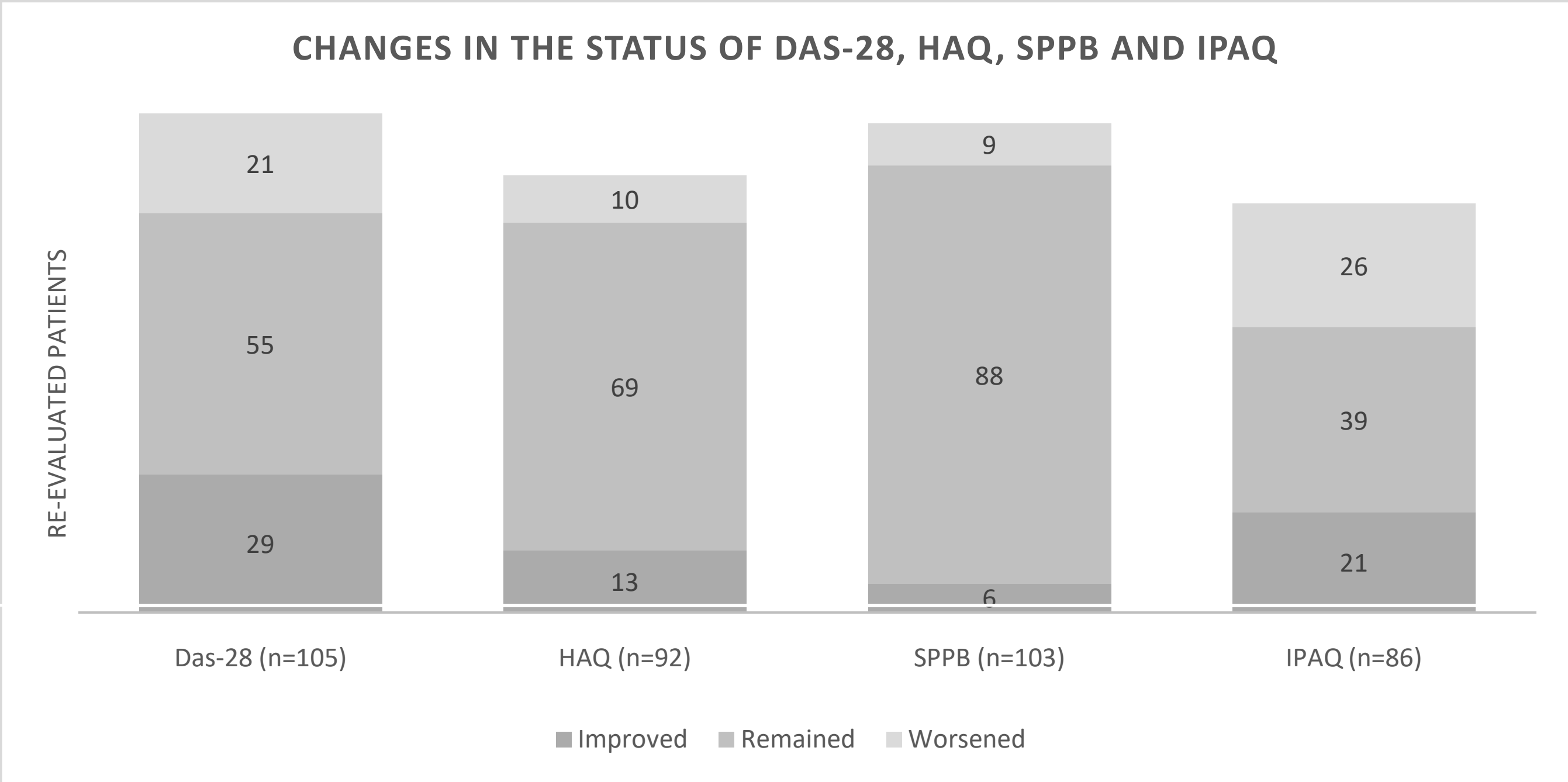

DAS-28: At baseline, among the re-evaluation patients, 42 were in remission, 19 were with low disease activity, 37 with moderate disease activity and 7 with higher disease activity.

HAQ: At baseline, among the re-evaluation patients, 47 had mild impairment, 35 had moderate impairment and 10 had severe impairment.

SPPB: At baseline, among the re-evaluation patients, 22 had low physical performance, and 81 had good physical performance.

IPAQ: At baseline, among the re-evaluation patients, 31 had low physical activity, 22 had moderate physical activity and 33 had high physical activity.

Test	Treatment	p
McNemar-Brwker	DAS-28	0.459
McNemar-Brwker	HAQ	0.709
McNemar test	SPPB	0.607
McNemar-Brwker	IPAQ	0.625
