# Supplementary material for: Muscle morphology changes over 1-year in patients with rheumatoid arthritis: a prospective cohort study using muscle ultrasound
Source: Adv Rheumatol. 2026 Jul 21;66:46. doi: 10.1186/s42358-026-00529-z (PMC13488837; doi:10.1186/s42358-026-00529-z)
Supplement: Supplementary file 2 [file 42358_2026_529_MOESM2_ESM.pdf]

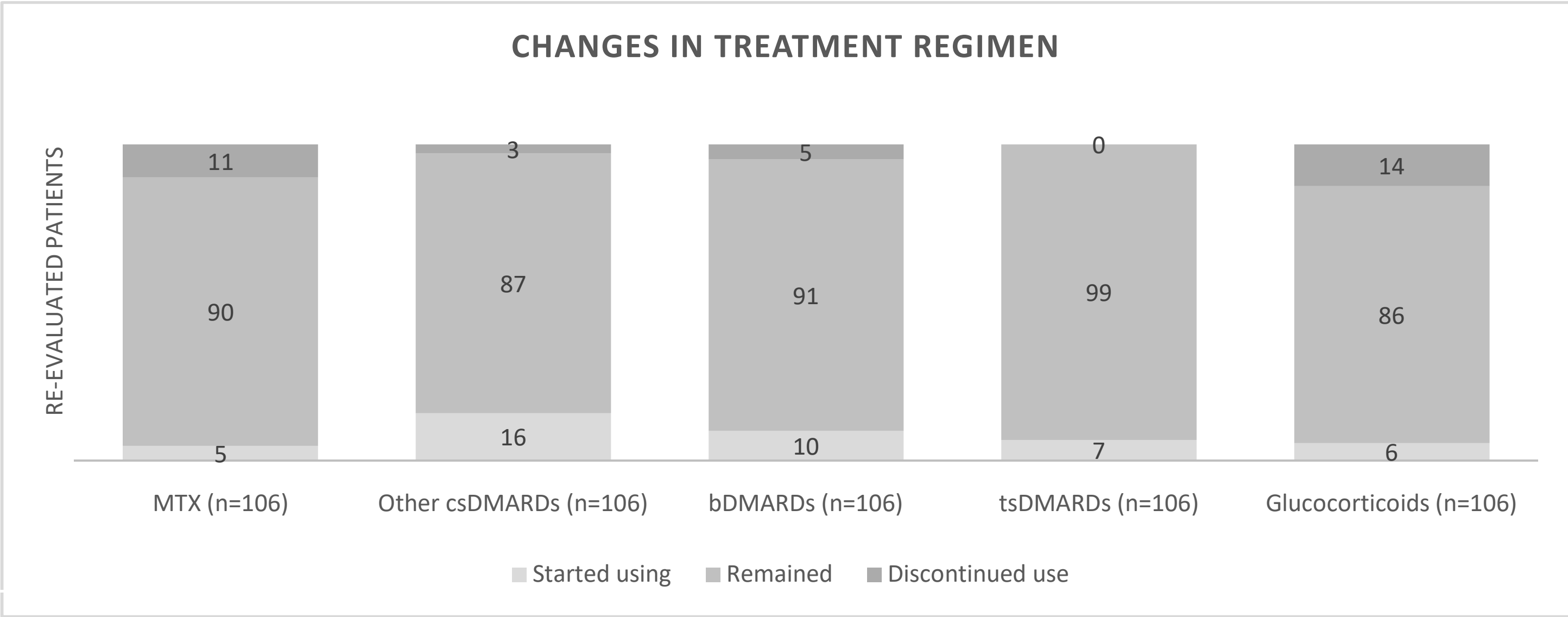

MTX: At baseline, 68 patients re-evaluation were using MTX and 38 were not using MTX.

Other csDMARDs: At baseline, 39 patients re-evaluation were using csDMARD and 67 were not using csDMARD.

bDMARDs: At baseline, 33 patients re-evaluation were using bDMARDs and 73 were not using bDMARDs.

tsDMARDs: At baseline, 6 patients re-evaluation were using tsDMARDs and 100 were not using tsDMARDs.

Glucocorticoids: At baseline, 30 patients re-evaluation were using glucocorticoids and 76 were not using glucocorticoids.

Abbreviation: MTX, Methotrexate; Other csDMARD, conventional synthetic disease-modifying antirheumatic drugs (Leflunomide, hydroxychloroquine, and sulfasalazine); bDMARDs, biologic disease-modifying antirheumatic drugs (adalimumab, etanercept, abatacept, infliximab, certolizumab, golimumab, rituximab, tocilizumab); tsDMARDS, target synthetic disease-modifying antirheumatic drugs (Tofacitinib, baracitinib);*MTX with or without glucocorticoids.

Test	Treatment	p, (all patients, n=106)	p, (no patients in remission, n=63)
McNemar-Brwker	MTX	0.210	0.065
McNemar-Brwker	csDMARD	0.004 *	0.004 *
McNemar-Brwker	bDMARD	0.302	0.388
McNemar-Brwker	tsDMARD	0.016 *	0.016 *
McNemar-Brwker	Glucocorticoids	0.115	0.388
