# Supplementary material for: Muscle morphology changes over 1-year in patients with rheumatoid arthritis: a prospective cohort study using muscle ultrasound
Source: Adv Rheumatol. 2026 Jul 21;66:46. doi: 10.1186/s42358-026-00529-z (PMC13488837; doi:10.1186/s42358-026-00529-z)
Supplement: Supplementary file 3 [file 42358_2026_529_MOESM3_ESM.pdf]

| | Performs physical exercise (n=81) |
|---|---|
| **Started training** | 5 |
| **Remained** | 71 |
| **Discontinued training** | 5 |
| **Total** | 81 |

| Test | p, (Exercise changes) |
|---|---|
| McNemar test | 1.000 |

**Physical exercises:** At baseline, among the re-evaluation patients (n=81), only 11 reported engaging in structured physical exercise at baseline (3 dance, 2 water-based exercise, and 6 resistance training).
At the 1-year follow-up, 15 patients reported exercising (1 dance, 12 resistance training, 1 physiotherapy, and 1 Pilates).
