# Supplementary material for: Muscle morphology changes over 1-year in patients with rheumatoid arthritis: a prospective cohort study using muscle ultrasound
Source: Adv Rheumatol. 2026 Jul 21;66:46. doi: 10.1186/s42358-026-00529-z (PMC13488837; doi:10.1186/s42358-026-00529-z)
Supplement: Supplementary file 4 [file 42358_2026_529_MOESM4_ESM.pdf]

ID	Baseline	1-year	Baseline treatment	Treatment modifications at 1 year	Treatment changes	
43	High disease activity	Low disease activity	MTX monotherapy	MTX **+ bDMARD**	Added bDMARD to treatment	#
67	High disease activity	Moderate disease activity	MTX + bDMARD + GC	**csDMARD** + bDMARD **sem GC**	Withdrew MTX and GC and added another csDMARD to treatment	#
81	High disease activity	Moderate disease activity	MTX + csDMARD + GC	csDMARD + GC	Withdrew MTX	#
82	High disease activity	Remission	MTX monotherapy	=	Treatment maintained	=
114	High disease activity	Moderate disease activity	MTX monotherapy	=	Treatment maintained	=
125	High disease activity	High disease activity	MTX + GC	MTX **+ tsDMARD** + GC	Added tsDMARD to treatment	#
126	High disease activity	Moderate disease activity	MTX + csDMARD + GC	csDMARD **+ bDMARD** + GC	Withdrew MTX and added bDMARD to treatment	#
2	Moderate disease activity	High disease activity	MTX + tsDMARD	=	Treatment maintained	=
6	Moderate disease activity	Moderate disease activity	MTX monotherapy	=	Treatment maintained	=
38	Moderate disease activity	Moderate disease activity	MTX + csDMARD + GC	MTX **+ bDMARD sem GC**	Withdrew csDMARD and GC and added bDMARD to treatment	#
39	Moderate disease activity	Remission	GC	**csDMARD + bDMARD sem GC**	Withdrew GC and initiated treatment with csDMARD and bDMARD	#
41	Moderate disease activity	Remission	csDMARD + GC	csDMARD **+ bDMARD** + GC	Added bDMARD to treatment	#
42	Moderate disease activity	Moderate disease activity	MTX + GC	Apenas MTX **sem GC**	Withdrew GC	#
44	Moderate disease activity	Remission	MTX monotherapy	MTX **+ tsDMARD**	Added tsDMARD to treatment	#
45	Moderate disease activity	High disease activity	GC	**MTX + csDMARD** + GC	Added MTX and another csDMARD to treatment	#
48	Moderate disease activity	Moderate disease activity	GC	**csDMARD sem GC**	Withdrew GC and initiated csDMARD	#
50	Moderate disease activity	Remission	MTX + csDMARD + GC	MTX **+ tsDMARD** + GC	Withdrew csDMARD and added tsDMARD to treatment	#
51	Moderate disease activity	Moderate disease activity	MTX monotherapy	**csDMARD + bDMARD**	Withdrew MTX and initiated another csDMARD and bDMARD	#
53	Moderate disease activity	Moderate disease activity	MTX + bDMARD	Apenas MTX	Withdrew bDMARD	#
58	Moderate disease activity	Moderate disease activity	tsDMARD + GC	=	Treatment maintained	=
59	Moderate disease activity	Low disease activity	MTX + bDMARD + GC	=	Treatment maintained	=
66	Moderate disease activity	Moderate disease activity	MTX monotherapy	**csDMARD**	Withdrew MTX and initiated another csDMARD	#
69	Moderate disease activity	Moderate disease activity	MTX monotherapy	**csDMARD**	Withdrew MTX and initiated another csDMARD	#
72	Moderate disease activity	Remission	csDMARD + bDMARD	=	Treatment maintained	=
91	Moderate disease activity	Moderate disease activity	csDMARD + bDMARD + GC	=	Treatment maintained	=
92	Moderate disease activity	Remission	MTX monotherapy	=	Treatment maintained	=
95	Moderate disease activity	Remission	MTX + bDMARD	bDMARD **+ GC**	Withdrew MTX and added GC to treatment	#
96	Moderate disease activity	Low disease activity	MTX + bDMARD + GC	MTX + GC	Withdrew bDMARD from treatment	#
103	Moderate disease activity	Remission	MTX + bDMARD	=	Treatment maintained	=
107	Moderate disease activity	Moderate disease activity	MTX + bDMARD	MTX + bDMARD **+ GC**	Added GC to treatment	#
115	Moderate disease activity	Low disease activity	csDMARD + bDMARD	=	Treatment maintained	=
116	Moderate disease activity	Moderate disease activity	MTX + bDMARD	Apenas MTX	Withdrew bDMARD from treatment	#
117	Moderate disease activity	Moderate disease activity	MTX monotherapy	MTX **+ GC**	Added GC to treatment	#
120	Moderate disease activity	Moderate disease activity	MTX + bDMARD	=	Treatment maintained	=
128	Moderate disease activity	Moderate disease activity	csDMARD + GC	=	Treatment maintained	=
133	Moderate disease activity	Remission	MTX monotherapy	MTX **+ csDMARD**	Added csDMARD to treatment	#
136	Moderate disease activity	Low disease activity	MTX monotherapy	MTX **+ tsDMARD**	Added tsDMARD to treatment	#

142	Moderate disease activity	Moderate disease activity	MTX monotherapy	MTX **+ csDMARD**	Added csDMARD to treatment	#
146	Moderate disease activity	Moderate disease activity	MTX + bDMARD	=	Treatment maintained	=
147	Moderate disease activity	Remission	csDMARD	=	Treatment maintained	=
148	Moderate disease activity	High disease activity	MTX + bDMARD	=	Treatment maintained	=
150	Moderate disease activity	Low disease activity	MTX monotherapy	csDMARD + bDMARD	Withdrew MTX and initiated csDMARD and Bdmard	#
155	Moderate disease activity	Moderate disease activity	MTX monotherapy	=	Treatment maintained	=
162	Moderate disease activity	Moderate disease activity	csDMARD + GC	csDMARD **+ bDMARD sem GC**	Withdrew GC and added bDMARD to treatment	#
7	Low disease activity	Low disease activity	MTX monotherapy	MTX **+ csDMARD**	Added csDMARD to treatment	#
25	Low disease activity	Remission	MTX + bDMARD	**csDMARD** + bDMARD	Withdrew MTX and added another csDMARD	#
30	Low disease activity	Remission	MTX + csDMARD	=	Treatment maintained	=
37	Low disease activity	Moderate disease activity	csDMARD	=	Treatment maintained	=
52	Low disease activity	Moderate disease activity	csDMARD + GC	csDMARD **+ tsDMARD** + GC	Added tsDMARD to treatment	#
60	Low disease activity	Remission	Apenas bDMARD	**MTX** + bDMARD	Added MTX to treatment	#
62	Low disease activity	Moderate disease activity	MTX + bDMARD + GC	=	Treatment maintained	=
64	Low disease activity	Moderate disease activity	Without treatment	=	Remained without treatment	=
65	Low disease activity	Remission	MTX + tsDMARD	=	Treatment maintained	=
70	Low disease activity	Moderate disease activity	MTX monotherapy	MTX **+ csDMARD**	Added csDMARD to treatment	#
75	Low disease activity	Remission	bDMARD	bDMARD + GC	Added GC to treatment	#
78	Low disease activity	Remission	csDMARD + bDMARD + GC	csDMARD + bDMARD **sem GC**	Withdrew GC from treatment	#
101	Low disease activity	Low disease activity	MTX + bDMARD	=	Treatment maintained	=
105	Low disease activity	Moderate disease activity	csDMARD + bDMARD + GC	csDMARD **+ tsDMARD** + GC	Switched bDMARD to tsDMARD	#
112	Low disease activity	Remission	MTX monotherapy	=	Treatment maintained	=
141	Low disease activity	Low disease activity	csDMARD + bDMARD	=	Treatment maintained	=
156	Low disease activity	Moderate disease activity	MTX + tsDMARD	=	Treatment maintained	=
157	Low disease activity	Moderate disease activity	MTX + tsDMARD	=	Treatment maintained	=
163	Low disease activity	Remission	MTX + GC	MTX **+ csDMARD + tsDMARD**	Withdrew GC and added another csDMARD and tsDMARD to treatment	#
10	Remission	Remission	MTX + csDMARD	=	Treatment maintained	=
11	Remission	Remission	MTX monotherapy	=	Treatment maintained	=
14	Remission	Remission	MTX + csDMARD	=	Treatment maintained	=
18	Remission	Remission	MTX monotherapy	=	Treatment maintained	=
24	Remission	Low disease activity	MTX monotherapy	=	Treatment maintained	=
40	Remission	Remission	MTX + bDMARD	=	Treatment maintained	=
46	Remission	Remission	csDMARD + bDMARD + GC	csDMARD + bDMARD **sem GC**	Withdrew GC from treatment	#
47	Remission	Remission	csDMARD	csDMARD **+ bDMARD**	Added bDMARD to treatment	#
54	Remission	Moderate disease activity	csDMARD + bDMARD + GC	csDMARD + bDMARD **sem GC**	Withdrew GC from treatment	#
55	Remission	Remission	csDMARD	=	Treatment maintained	=
57	Remission	Low disease activity	MTX monotherapy	=	Treatment maintained	=
61	Remission	Remission	MTX monotherapy	=	Treatment maintained	=

63	Remission	Low disease activity	bDMARD	**MTX** + bDMARD	Added MTX to treatment	#
76	Remission	Remission	MTX monotherapy	=	Treatment maintained	=
77	Remission	Remission	Without treatment	**MTX + csDMARD**	Initiated treatment with MTX and csDMARD	#
79	Remission	Remission	csDMARD + bDMARD	=	Treatment maintained	=
84	Remission	Moderate disease activity	MTX + GC	MTX **sem GC**	Withdrew GC from treatment	#
94	Remission	Remission	MTX + bDMARD	=	Treatment maintained	=
98	Remission	Remission	MTX + csDMARD	=	Treatment maintained	=
99	Remission	Remission	csDMARD	=	Treatment maintained	=
100	Remission	Remission	MTX monotherapy	=	Treatment maintained	=
102	Remission	Remission	csDMARD	=	Treatment maintained	=
109	Remission	Remission	csDMARD + bDMARD	=	Treatment maintained	=
110	Remission	Remission	csDMARD + bDMARD + GC	=	Treatment maintained	=
113	Remission	Remission	MTX + GC	MTX **+ bDMARD sem GC**	Withdrew GC and added bDMARD to treatment	#
118	Remission	Remission	csDMARD + bDMARD	csDMARD **+ GC**	Withdrew bDMARD and added GC to treatment	#
121	Remission	Remission	csDMARD	=	Treatment maintained	=
122	Remission	Remission	csDMARD	=	Treatment maintained	=
123	Remission	Remission	MTX + csDMARD	**Apenas csDMARD**	Withdrew MTX from treatment	#
129	Remission	Moderate disease activity	csDMARD + tsDMARD	**Apenas tsDMARD**	Withdrew csDMARD from treatment	#
131	Remission	Low disease activity	MTX monotherapy	=	Treatment maintained	=
132	Remission	Low disease activity	MTX monotherapy	=	Treatment maintained	=
134	Remission	Remission	MTX + csDMARD	=	Treatment maintained	=
135	Remission	Remission	MTX + bDMARD	=	Treatment maintained	=
140	Remission	Low disease activity	csDMARD + GC	csDMARD **sem GC**	Withdrew GC from treatment	#
143	Remission	Remission	csDMARD	=	Treatment maintained	=
152	Remission	Remission	MTX monotherapy	=	Treatment maintained	=
153	Remission	Remission	MTX + bDMARD	=	Treatment maintained	=
154	Remission	Remission	csDMARD	=	Treatment maintained	=
158	Remission	Remission	MTX + GC	MTX **sem GC**	Withdrew GC from treatment	#
160	Remission	Moderate disease activity	GC	**MTX + csDMARD** + GC	Added MTX and csDMARD to treatment	#
161	Remission	Remission	MTX + csDMARD	=	Treatment maintained	=
119	Unavailable data	Moderate disease activity	MTX monotherapy	**GC**	Withdrew MTX and added GC only	#

Abbreviation: MTX, Methotrexate; csDMARD, conventional synthetic disease-modifying antirheumatic drugs (methotrexate, leflunomide, hydroxychloroquine, and sulfasalazine); bDMARDs, biologic disease-modifying antirheumatic drugs (adalimumab, etanercept, abatacept, infliximab, certolizumab, golimumab, rituximab, tocilizumab); tsDMARDS, target synthetic disease-modifying antirheumatic drugs (Tofacitinib, baracitinib); GC, glucocorticoids; *MTX with or without glucocorticoids; #, Changed; =, Same.
